# Work-Related Illnesses and Work Disability in Brazil: A Scoping Review of the Epidemiological Profile and Determinants of Sick Leave and Occupational Accidents

**DOI:** 10.3390/epidemiologia7040111

**Published:** 2026-08-14

**Authors:** Luciano Garcia Lourenção, Luciano Silveira Pacheco de Medeiros

**Affiliations:** 1 Office of the Minister of State, Ministry of Social Security, Brasília 70059-900, DF, Brazil; 2 School of Nursing, Federal University of Rio Grande, Rio Grande 96200-400, RS, Brazil; lucianospm@outlook.com

**Keywords:** sick leave, accidents, occupational, occupational diseases, health profile, social security

## Abstract

**Background:** This scoping review maps and synthesizes the scientific evidence on the epidemiological profile and determinants of sick leave and work-related accidents in Brazil, with an emphasis on studies using data from the Brazilian Federal Medical Expert Service of the Ministry of Social Security. **Methods:** Following the PRISMA-ScR guidelines and the JBI Manual for Evidence Synthesis, eight databases (SciELO, PubMed/MEDLINE, Scopus, Web of Science, LILACS, CINAHL, PsycINFO, Embase) were searched for quantitative observational studies published between 2000 and 2025. Out of the 218 studies identified, 18 met the eligibility criteria. **Results:** Cross-sectional designs (66.7%) and ecological/time-series designs (22.2%) predominated. The findings identified musculoskeletal disorders as the leading cause of work absence; mental disorders were the third leading cause, exhibiting an upward trend; and a significant influence of institutional factors, especially the Social Security Technical Epidemiological Nexus, on the observed patterns. Determinants operate at the individual, occupational/organizational, and institutional levels. **Conclusions:** Empirical research on work disability in Brazil remains fragmented, providing limited capacity for causal inference. The findings support five priority areas: sectoral risk management, workplace mental health, territorial strategies for musculoskeletal disorders, sick leave trajectory policies, and data governance, all of which necessitate longitudinal and quasi-experimental studies to evaluate interventions and the impact of institutional changes.

## 1. Introduction

Work disability is a major public health and social protection issue, reflecting the intersection of working conditions, pathological processes, and institutional mechanisms for disability assessment [1]. Sick leave and workplace accidents directly impact the economically active population, compromising productive capacity, workers’ income, and the sustainability of social security systems [1].

The impact of musculoskeletal conditions on sickness absenteeism has been the subject of systematic reviews that identify multiple prognostic factors, underscoring the complexity of the phenomenon and the need for integrated approaches [2]. In Brazil, this phenomenon is particularly significant due to the magnitude of disability benefits granted annually and their socioeconomic and health effects on individuals, families, corporations, and public policies [3].

In the Brazilian Social Security system, distinct concepts must be clarified. Sick leave refers to temporary disability benefits granted to workers unable to perform their usual activities [4]. These are classified as non-work-related (B31) or work-related/accidentary (B91), depending on whether an occupational causal link is established [5,6]. Work-related accidents are events that may generate B91 benefits [4]. Work disability is the broader concept encompassing all situations in which a worker’s health condition prevents work, as assessed by the Federal Medical Expert Service [7]. The Social Security Technical Epidemiological Nexus (NTEP) is the institutional mechanism that establishes the causal link between the health condition and the work activity [8,9,10].

In this review, “determinants” refer to the sociodemographic (sex, age, education, geographic region), occupational (economic sector, occupation), and institutional (benefit type, NTEP application) factors that can be operationalized from administrative and medical expert records available in Brazilian Social Security databases. Psychosocial, lifestyle, and organizational factors are discussed as theoretical background but were not directly analyzed, as they fall outside the scope of administrative data [11,12].

In recent decades, an epidemiological transition has also been observed in the occupational landscape, marked by a relative reduction in classic work-related injuries associated with manual labor and an increase in chronic diseases, musculoskeletal disorders, and mental disorders as the predominant causes of sickness absenteeism, often distributed unevenly across economic sectors, regions, and demographic groups [13]. In this transition, psychosocial occupational exposures have been systematically associated with the development of common mental disorders, constituting a relevant explanatory framework for understanding the rise in work absences due to mental disorders [14]. Recent evidence further suggests that sickness absence is not merely a clinical outcome but the result of complex pathways involving psychosocial work factors and individual well-being [15]. This multidimensional nature of work disability is also influenced by sex-specific associations between lifestyle factors and absenteeism [16], as well as the long-term impact of prior socioeconomic and health history on later disability trajectories [17]. Although these factors are widely documented in the international literature, the present scoping review focuses specifically on how they have been investigated using Brazilian Social Security administrative and medical expert data. This background provides the conceptual foundation for the review’s analytical framework.

In this context, the Brazilian Social Security system plays a strategic role as the primary safety net for formal workers, while also serving as a valuable source of epidemiological data on work disability [1]. The administrative and medical records produced under the General Social Security System allow for the monitoring, on a national scale, of patterns of sick leave due to illness and work-related accidents, as well as the granting of benefits for temporary and permanent disability [3]. The Federal Medical Expert Service, as the body responsible for the assessment and institutional recognition of disability, plays a central role in this process by mediating the relationship between illness, work, and social protection, including the characterization of the causal link between health conditions and work activities [7].

Starting in the 2000s, Brazil saw an expansion of social security coverage, the reorganization of disability benefits, and the progressive strengthening of national health and labor information systems, which enabled greater systematization and stability of the administrative records of the National Social Security Institute [1]. Throughout the 2000s, these advances enabled the production of more consistent historical series and the conduct of more comprehensive epidemiological analyses of sick leave, work-related accidents, and the granting of social security benefits [1]. The implementation of the Social Security Technical Epidemiological Nexus in 2007–2008 represented a significant turning point by expanding institutional recognition of the relationship between work and illness. However, changes in observed benefit patterns may also reflect modifications in administrative procedures and disability assessment practices, rather than solely changes in morbidity.

Starting in 2015, the Brazilian social security system underwent a series of far-reaching institutional and regulatory changes, including the reorganization of the Federal Medical Expert Service, the digitalization and standardization of medical assessment processes, and the intensification of disability benefit reviews [7]. These changes culminated in the 2019 Social Security Reform, which altered the criteria for accessing, calculating, and maintaining benefits, with potential repercussions on the profile of sick leaves and the recognition of work disability [7]. More recently, the COVID-19 pandemic introduced an exceptional scenario, characterized by massive sick leave, the adoption of remote medical examinations, and temporary changes in medical assessment criteria, followed by a process of institutional reorganization and normalization of workflows between 2023 and 2025 [18].

Although studies addressing sick leave and work-related accidents in Brazil exist, they are predominantly focused on specific conditions, isolated economic sectors, or regional settings. This fragmented landscape limits the capacity to correlate the epidemiological profile of sick leave with its occupational determinants and the institutional transformations of the social security system [1,19].

Given this scenario, the following research question arises: how does the scientific literature characterize the epidemiological profile and determinants of sick leave due to illness and work-related accidents in Brazil, based on studies using administrative and medical assessment data from Social Security, from 2000 to 2025? Based on this question, the present study aims to map and synthesize the scientific evidence on the epidemiological profile and determinants of sick leave and work-related accidents in Brazil, with an emphasis on studies using data from the Federal Medical Expert Service.

## 2. Materials and Methods

### 2.1. Study Design

This scoping review was conducted in accordance with the Preferred Reporting Items for Systematic Reviews and Meta-Analyses extension for Scoping Reviews (PRISMA-ScR) guidelines. The study followed a predefined protocol registered in the Open Science Framework (OSF) (DOI: https://doi.org/10.17605/OSF.IO/CM95X). A completed PRISMA-ScR checklist is provided as Supplementary Materials (PRISMA 2020 Checklist).

The review followed the methodological steps recommended for this design [20]: (a) formulation of the research question; (b) systematic identification of potentially relevant studies; (c) selection of studies according to explicit eligibility criteria; (d) mapping and extraction of key information; and (e) synthesis and presentation of results in a descriptive and analytical manner. The search strategy was structured using the PCC acronym (Population, Concept, and Context) [21], from which the following guiding question was formulated: “How does the scientific literature characterize the epidemiological profile and determinants of sick leave and work-related accidents in Brazil, based on studies using administrative and expert assessment data from Social Security, from 2000 to 2025?”

In this design, the Population (P) included adult workers (≥18 years old) formally enrolled in the Brazilian social security system; the Concept (C) encompassed the epidemiological profile and the sociodemographic, occupational, organizational, and institutional determinants of sick leave, work-related accidents, and the granting of disability benefits, as investigated in the literature using administrative and medical expert assessment data; and the Context (C) corresponded to the Brazilian social security system, with an emphasis on studies based on administrative and medical expert records from the General Social Security System, especially those produced or systematized by the Federal Medical Expert Service as the institutional body responsible for assessing work disability.

### 2.2. Data Collection

The search strategy was developed systematically and conducted in December 2025. Initially, an exploratory phase was conducted by consulting the PROSPERO, Cochrane Database of Systematic Reviews, and JBI Evidence Synthesis platforms to identify ongoing or recently published systematic or scoping reviews on the topic, verifying possible overlaps and avoiding duplication of research efforts. No ongoing or recently published reviews with an identical or substantially overlapping scope were identified; therefore, the review proceeded as planned.

Following this preliminary stage, a structured search of the scientific literature was conducted in the following electronic databases: SciELO, PubMed/MEDLINE, Scopus, Web of Science, LILACS, CINAHL, PsycINFO, and Embase. These databases were selected for their broad multidisciplinary coverage spanning public health, occupational health, epidemiology, social sciences applied to health, and social security policies, allowing for greater sensitivity in identifying studies related to work disability, sick leave, and workplace accidents in the Brazilian context.

**Figure 1:**
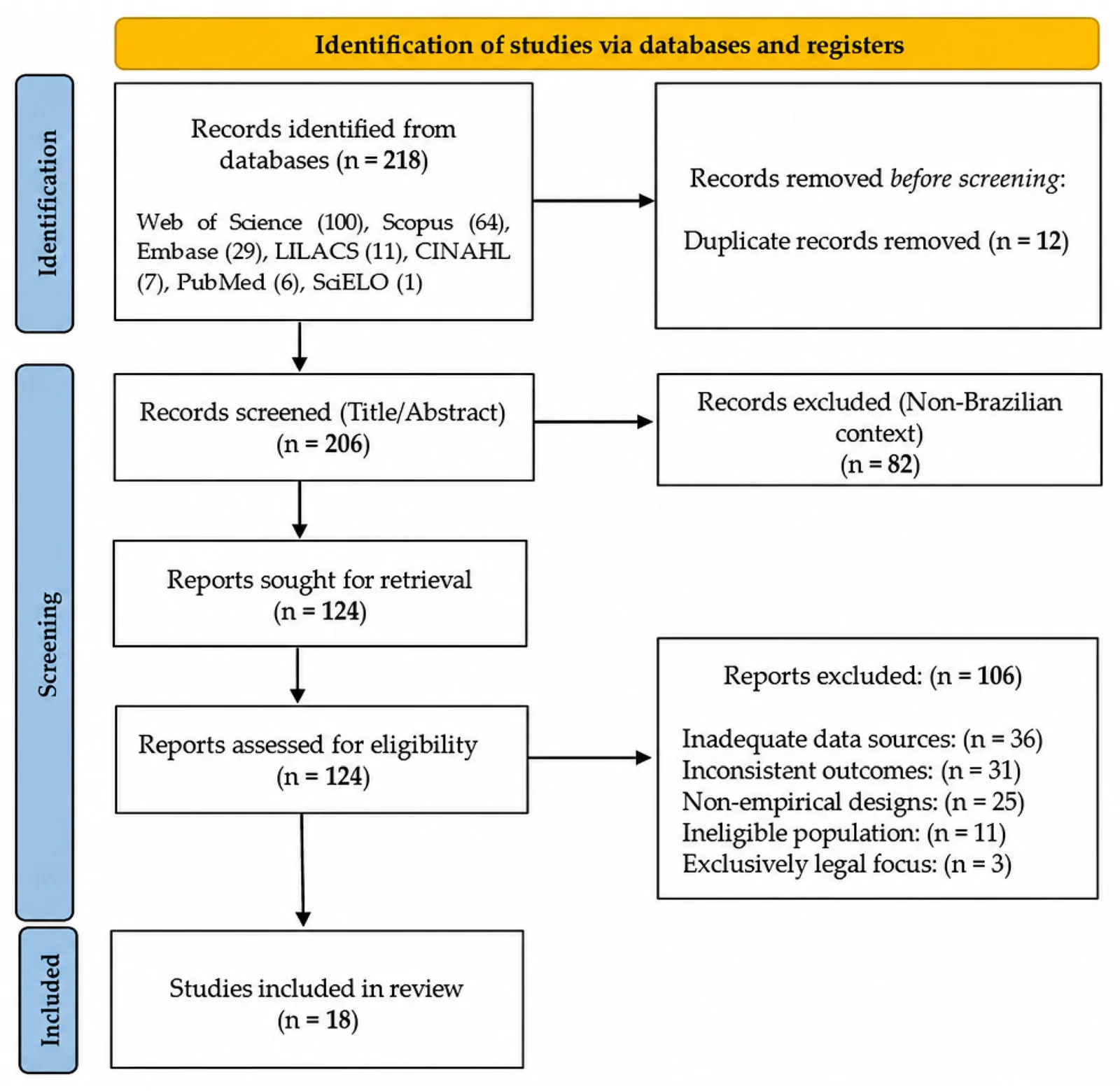
Flowchart of the process of identification, screening, eligibility, and inclusion of studies. Note: Studies were excluded after screening of titles and abstracts when they did not meet the eligibility criteria. Reasons for exclusion included (1) non-Brazilian context, (2) inadequate data source (clinical, self-reported, institutional, or absenteeism data, without use of Social Security administrative/expert databases), (3) incompatible outcome (work absenteeism, symptoms, or diseases without analysis of disability benefits), (4) ineligible population or employment relationship (outside the General Social Security System), (5) non-empirical study type, and (6) legal focus, without epidemiological analysis of social security.

Study selection followed a two-stage process, in accordance with the PRISMA-ScR guidelines (Item 8). In the first stage, titles and abstracts of all 206 non-duplicate records were screened independently by two reviewers (L.G.L. and L.S.P.M.) against the eligibility criteria. Records that clearly did not meet the criteria were excluded. In the second stage, the full text of the remaining 124 studies was assessed independently by the same two reviewers. Disagreements at both stages were resolved by consensus discussion. The selection process is documented in the PRISMA flowchart (Figure 1), and the reasons for full-text exclusion are listed in Section 3.1.

Data extraction was performed by one reviewer (L.S.P.M.) using a pre-structured electronic form developed in Microsoft Excel, covering (a) study identification (authors, year, journal); (b) methodological characteristics (design, population, sector, data source, period); (c) main findings related to sick leave, work-related accidents, and work disability; and (d) institutional factors addressed. The extracted data were independently verified for accuracy by a second reviewer (L.G.L.). Discrepancies were resolved by consensus. This process aligns with the PRISMA-ScR guidelines (Item 9).

### 2.3. Search Strategy

The search strategy was developed systematically by combining controlled vocabulary terms and free-text terms related to work disability, sick leave, workplace accidents, and the social security system, in accordance with the PCC framework. The following terms were used: (“Work Disability” OR “Incapacidade para o Trabalho” OR “Work Ability” OR “Sickness Absence” OR “Sickness Absenteeism”) AND (“Occupational Diseases” OR “Doença Ocupacional” OR “Occupational Accidents” OR “Acidentes de Trabalho”) AND (“Social Security” OR “Social Insurance” OR “Previdência Social”) AND (Brazil OR Brasil).

To ensure greater sensitivity and coverage, descriptors from the Health Sciences Descriptors (DeCS) and Medical Subject Headings (MeSH) were used, combined with free-text terms widely used in the literature. Controlled descriptors included Work Disability, Sickness Absence, Occupational Diseases, Occupational Accidents, and Social Security, as well as their Portuguese equivalents. Boolean operators (AND, OR) were used strategically to expand the retrieval of potentially eligible studies while respecting the specific indexing criteria of each database. The search strategy was adapted for each database to comply with specific indexing syntaxes, field codes, and controlled vocabulary requirements. For databases that do not use MeSH or DeCS indexing (e.g., Scopus, Web of Science, Embase), controlled vocabulary terms were replaced by equivalent free-text terms and database-specific subject headings (e.g., Emtree terms for Embase), combined with Boolean operators to maintain search sensitivity. The complete adapted strings for each database are provided as Supplementary Materials (Search Strategies by Database).

### 2.4. Eligibility Criteria

The eligibility criteria were defined to include empirical studies directly related to the epidemiological profile and determinants of sick leave and work-related accidents in Brazil, based on records from the Federal Medical Expert Service and Social Security. We included quantitative observational studies (cross-sectional, cohort, case–control, ecological, and time-series studies) based on secondary data from administrative, medical expert, and social security sources—including records from the Federal Medical Expert Service, the Disability Benefits Administration System (Sistema de Administração de Benefícios por Incapacidade—SABI), Social Security Information and Technology Enterprise (Empresa de Tecnologia e Informações da Previdência—DATAPREV), and National Social Security Information Registry (Cadastro Nacional de Informações Sociais—CNIS)—involving adult workers (≥18 years) formally enrolled in the Brazilian social security system.

Studies were considered eligible if they addressed at least one of the following outcomes, provided they also presented data on the epidemiological profile or determinants: temporary or permanent work disability, sick leave due to illness, work-related accidents, granting of disability benefits, or characterization of the work-illness link. The time frame included publications from 2000 to 2025 in Portuguese, English, or Spanish.

Non-empirical studies with an exclusively legal, clinical, or therapeutic focus were excluded, as were those based solely on self-reports without institutional validation. Also excluded were studies involving populations without social security coverage, informal workers, children, or adolescents; studies restricted to short-term absenteeism (<15 days); and studies whose data sources did not include official administrative, medical, or social security records.

### 2.5. Data Synthesis and Analysis

After defining the final corpus, relevant information was systematically extracted using a pre-structured form, covering methodological characteristics (year of publication, study design, population, occupational sector, and data source), as well as the main findings related to sick leave, work-related accidents, and work disability. The narrative synthesis followed a three-step process: (1) studies were grouped by methodological design and temporal period; (2) thematic categories were developed iteratively based on recurring patterns in the extracted data (epidemiological profile, determinants, institutional factors); (3) within each category, findings were compared across studies to identify convergences, divergences, and gaps. The categories were refined through discussion between the two reviewers. The synthesis was conducted manually, organized in Microsoft Excel (Microsoft 365, version 16.0) spreadsheets for thematic categorization and comparison across studies. No qualitative analysis software was used, given the quantitative nature of the included studies and the descriptive objective of the review.

The institutional milestones presented in Section 3.2 (NTEP implementation, the 2019 Social Security Reform, and the COVID-19 pandemic) are contextual references used to organize the temporal distribution of the included studies, not variables analyzed in the synthesis. They are derived from the reviewed literature and institutional documents cited in the Introduction.

In accordance with the Joanna Briggs Institute (JBI) and PRISMA-ScR guidelines for scoping reviews, a formal critical appraisal of the risk of bias of the included studies was not performed, as the primary objective of this review is to map the breadth and nature of the evidence rather than to assess the methodological quality or certainty of individual study results.

The synthesis focused on providing a comprehensive overview of the morbidity profile and epidemiological landscape of occupational health in Brazil. Specific effect measures, such as risk ratios, odds ratios, or mean differences, were not used in the synthesis or presentation of results due to the descriptive and narrative nature of this scoping review. Consequently, no methods for assessing reporting bias or the certainty of evidence (e.g., GRADE) were applied.

The final results are presented through descriptive tables and figures and a qualitative discussion that integrates the findings with the current public policies and social security regulations in Brazil.

## 3. Results

### 3.1. Study Selection

A total of 218 studies were identified across electronic databases, including 100 (45.9%) from Web of Science, 64 (29.4%) from Scopus, 29 (13.3%) from Embase, 11 (5.0%) from LILACS, 7 (3.2%) from CINAHL, 6 (2.8%) from PubMed/MEDLINE, and 1 (0.5%) from SciELO. After removing 12 duplicate records (5.5%), 206 studies (94.5%) remained for the title and abstract screening phase. In the initial screening, 82 studies (39.8%) were excluded for addressing non-Brazilian contexts, resulting in 124 studies (60.2%) eligible for full-text review.

During the full-text evaluation stage, 106 studies (85.5%) were excluded for failing to meet the eligibility criteria. The main reasons for exclusion were inadequate data sources (36 studies; 29.0%), outcomes inconsistent with the review’s objective (31 studies; 25.0%), non-empirical study designs (25 studies; 20.2%), ineligible population or social security affiliation (11 studies; 8.9%), and an exclusively legal focus without epidemiological analysis (3 studies; 2.4%). At the end of the selection process, 18 studies (8.3% of the total initially identified) met all eligibility criteria and were included in the final synthesis, as shown in the PRISMA flowchart (Figure 1). The characteristics of the studies are presented in Supplementary Materials (Table S1).

### 3.2. Temporal Distribution and Institutional Milestones

#### 3.2.1. Temporal Distribution of Included Studies

**Figure 2:**
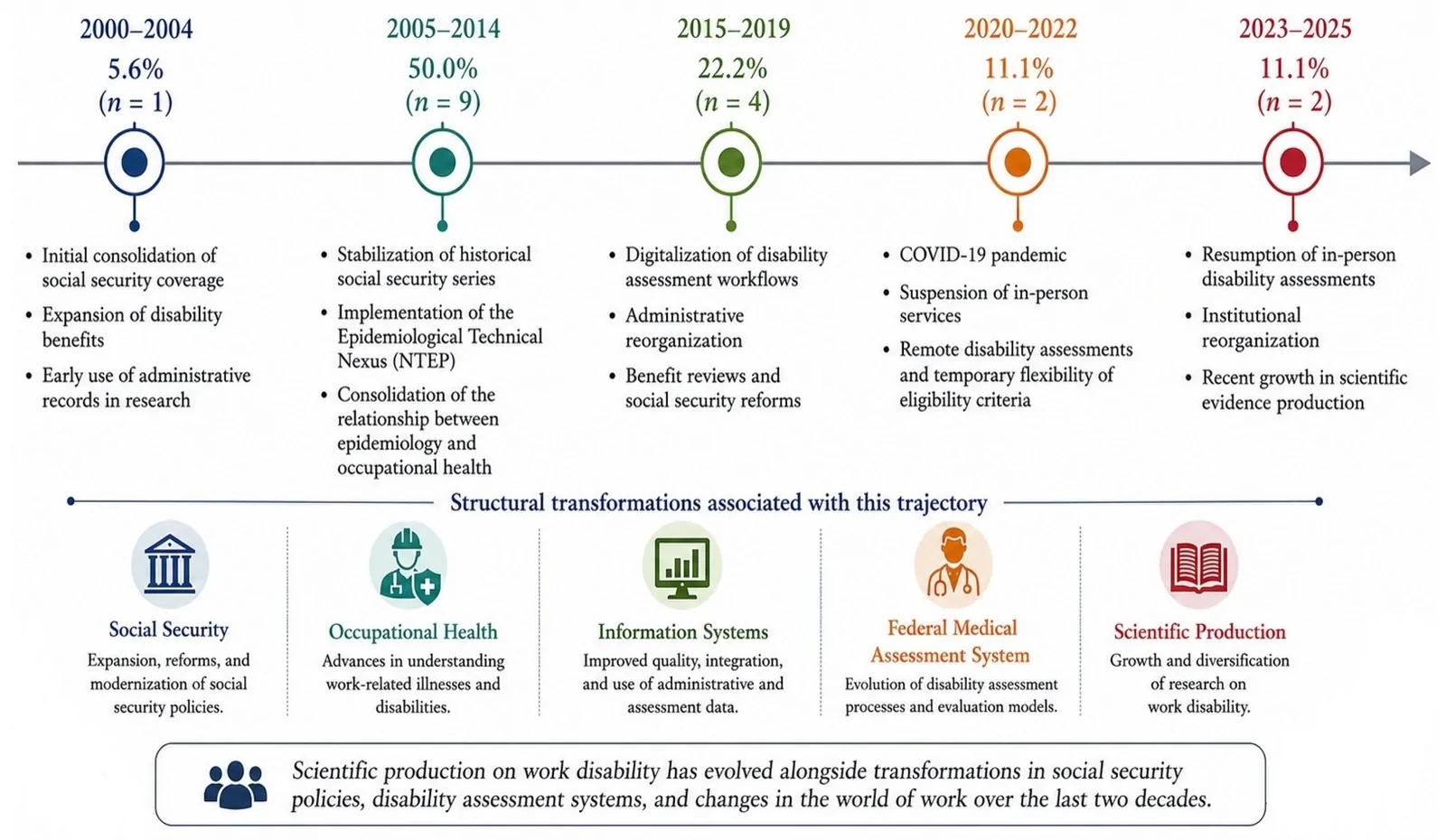
Distribution of included studies by time period (2000–2025).

The temporal distribution of the studies (Figure 2) shows that research was distributed across several periods, with one study published between 2000 and 2004 (5.6%) but most concentrated in the period from 2005 to 2014 (50.0%), followed by 2015–2019 (22.2%), 2020–2022 (11.1%), and 2023–2025 (11.1%).

#### 3.2.2. Institutional Context

The institutional phases described are presented as contextual background to aid interpretation of the temporal distribution of studies (Figure 2). They correspond to the analytical periods adopted in the broader research program of which this review is part and are not findings derived from the included studies. Between 2000 and 2004, the use of Social Security administrative records was in its early stages [22]. The period from 2005 to 2014 followed the stabilization of historical time series and the implementation of the Social Security Technical Epidemiological Nexus, accompanied by the consolidation of the epidemiological transition, marked by an increase in chronic diseases, musculoskeletal disorders, and mental disorders as causes of sick leave [6,23,24,25,26,27,28,29,30].

Between 2015 and 2019, notable institutional changes included administrative reorganization, the digitalization of medical expert workflows, and the intensification of benefit reviews [31,32,33,34]. The period from 2020 to 2022 was characterized by exceptional changes resulting from the COVID-19 pandemic, with the suspension of in-person medical expert assessments and the adoption of remote assessments [10,35]. Finally, between 2023 and 2025, a restoration of assessment flows and institutional normalization was observed, accompanied by recent evidence on the epidemiological profile of work disability in the country [1,3].

### 3.3. Methodological Characteristics

**Figure 3:**
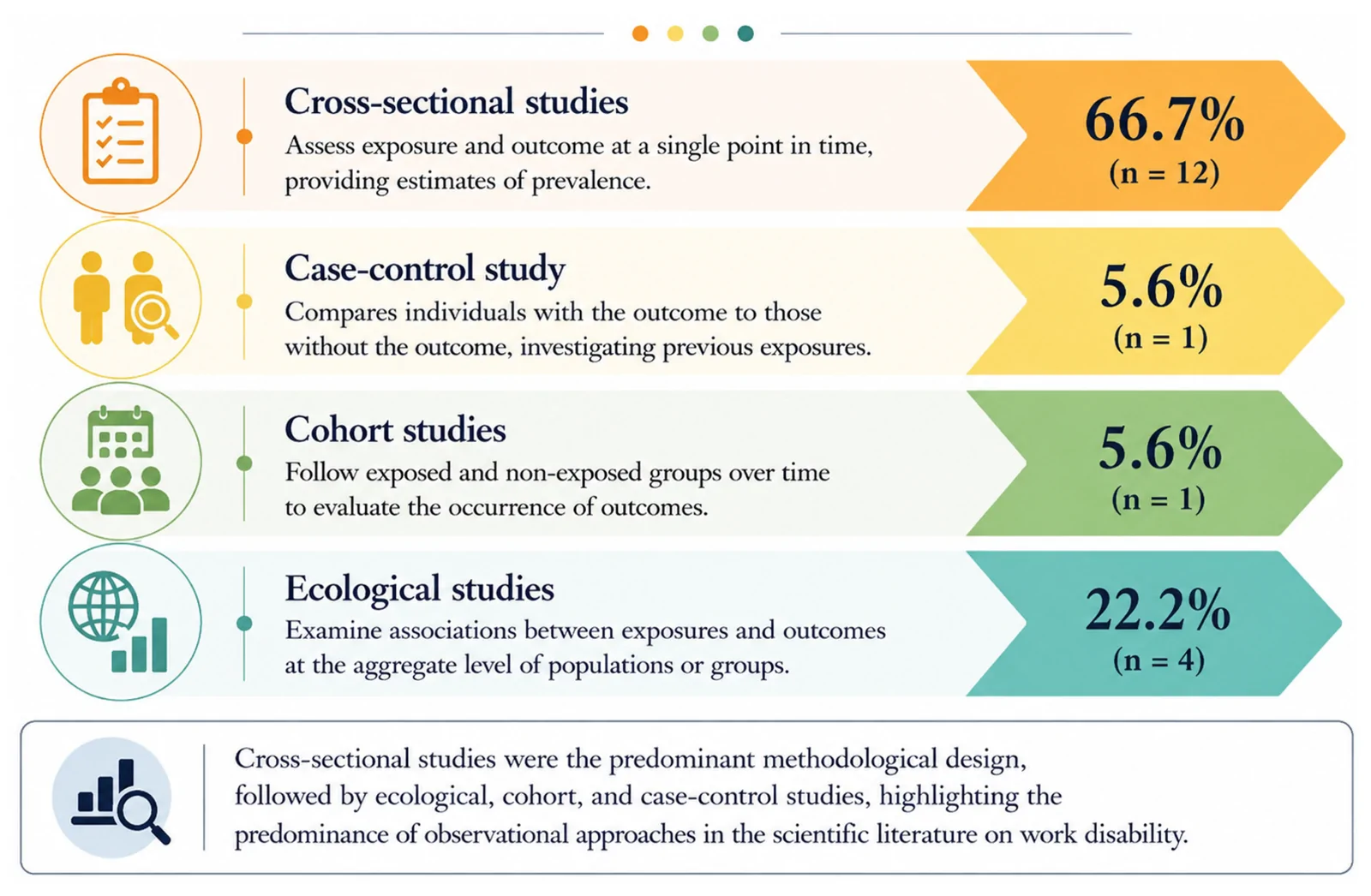
Distribution of selected studies by study design.

Analysis of the methodological designs of the selected studies (Figure 3) revealed a predominance of cross-sectional studies, which accounted for 66.7% of the corpus (12 studies), reflecting the extensive use of administrative and social security data to characterize the epidemiological profile of disability-related leave. Next were ecological studies, including time series, which accounted for 22.2% (4 studies), focusing particularly on the analysis of temporal trends and the impact of regulatory and institutional changes on the social security system.

Cohort and case–control studies were equally infrequent, with only one study for each design (5.6% each), indicating a lower availability of longitudinal analyses. This methodological pattern highlights the descriptive nature of the available literature, which is sufficient for mapping patterns and inequalities but limited for robust causal inferences regarding the determinants of work disability in Brazil.

### 3.4. Epidemiological Profile and Determinants

The epidemiological profile of work-related absences in Brazil reveals a shift from predominantly infectious causes to chronic non-communicable diseases (CNCDs) and conditions related to work organization [22], with temporary disability concentrated particularly among private-sector workers enrolled in the General Social Security System, specifically in the 30–59 age group [6,30].

Disability-related morbidity is led by Work-Related Musculoskeletal Disorders (WMSDs), which account for the highest burden of sick leave, with a high prevalence of low back pain, synovitis, and tenosynovitis [30,31]. High prevalence rates are notably concentrated in the Southeast and South regions, which encompass Brazil’s most industrialized and productive centers, with localized evidence indicating an upward trend in these areas [10,30]. These disorders primarily affect women and workers subjected to biomechanical overload [30,31].

Mental and behavioral disorders, which have become the third leading cause of social security benefits [1,29], showed an increase in severity in the post-COVID-19 pandemic period, particularly depressive episodes and anxiety disorders [3]. External causes and respiratory/cardiovascular diseases, although less frequent than WMSDs, are typically associated with longer work absences (especially cardiovascular diseases and neoplasms) and generate high social security costs [22,25,33,35]. The main findings, stratified by research domain, are summarized in Table 1.

The factors associated with these absences are multifactorial, extending beyond individual biology and pointing to socioeconomic and occupational dimensions [1,30]. Individual and social factors, such as female gender, advanced age, and low educational attainment, are identified as recurring predictors of the occurrence of work-related leave [23,26,30,31]. However, regarding duration, low socioeconomic status and age below 39 years are associated with higher rates of benefit cessation, resulting in shorter absences and a faster return to work, while no significant differences in benefit duration have been found regarding sex [23,27] (Table 2).

At the same time, organizational and psychosocial factors, including Job Strain (Karasek’s Model), Effort-Reward Imbalance (Siegrist’s Model), repetitiveness, and poor posture, are considered central to mental illness and the link to WMSDs [29,30,34]. At the institutional level, the implementation of the Social Security Technical Epidemiological Nexus (NTEP) represents an important milestone in expanding recognition of the causal link and reducing historical underreporting [1,10], although underreporting persists in sectors with high levels of informality [30].

## 4. Discussion

This scoping review identified a relevant yet fragmented body of evidence to inform public health and social protection decisions. Although 218 studies were retrieved, only 18 met the eligibility criteria, indicating that empirical research utilizing social security and medical expert data to characterize illness and work disability remains concentrated on specific conditions, geographic areas, and economic sectors.

From a methodological standpoint, the predominance of cross-sectional and ecological/time-series designs, with a scarcity of cohort studies, is consistent with the nature of administrative databases—offering high coverage for monitoring but lower causal explanatory power. This has two critical implications: (i) the available literature is sufficient to delineate patterns and inequalities (who becomes ill, where, and in which sectors), but (ii) it is less capable of rigorously explaining the underlying mechanisms of these patterns or identifying which interventions most effectively reduce the risk and duration of sick leave. The predominance of cross-sectional designs limits causal inference and the evaluation of interventions. Future research should prioritize longitudinal and quasi-experimental designs, such as interrupted time series analysis, that enable the distinction between real changes in illness patterns and effects resulting from institutional and regulatory transformations.

The results show a concentration of studies in the 2005–2014 period, coinciding with the stabilization of historical series and the implementation of the Social Security Technical Epidemiological Nexus. This milestone is pivotal because disability benefit series reflect not only the occurrence of illness but also the institutional mechanisms for recognizing occupational causality and producing forensic data. Changes in observed benefit patterns following the NTEP may result from a combined effect: (a) actual changes in morbidity and occupational risk, (b) expanded institutional recognition of the work-illness link, and (c) modifications in administrative procedures and disability assessment practices. The available evidence does not allow these effects to be fully disentangled, a limitation that underscores the need for study designs capable of distinguishing epidemiological from institutional change [1,10].

The results point to systemic transformations during the 2015–2019 period, including the reorganization of the Federal Medical Expert Service, the digitalization of workflows, and the intensification of benefit reviews, culminating in the 2019 Social Security Reform [1,3]. A study based on bibliographic and documentary research demonstrated that this reform introduced more restrictive criteria for accessing and maintaining benefits, with the potential to significantly alter historical flows [7]. Subsequently, between 2020 and 2022, the COVID-19 pandemic introduced an operational and epidemiological disruption, marked by the suspension of in-person assessments and the adoption of remote medical assessment criteria that preclude direct comparability with previous historical series [1,3]. This scenario was further characterized by a transition to digital platforms that, while seeking efficiency, may have imposed additional barriers on workers with limited technological access [7].

Finally, the recovery between 2023 and 2025 reintroduces the need for recent analyses capable of distinguishing “real epidemiological change” from “institutional/regulatory change,” a distinction reinforced by recent studies on occupational mental disorders and WMSDs from a spatiotemporal perspective [1,13]. Thus, the findings should not be interpreted as a homogeneous continuous line, but rather as a sequence of institutional regimes that modulate the statistical visibility of the phenomenon.

The studies reinforce that work disability, when observed through temporary benefits, is a multicausal phenomenon with a strong component of chronicity and recurrence. The analyzed content converges on three areas of programmatic relevance: (i) mental disorders as a critical axis of disabling leave [1,28,29,34]; (ii) Work-Related Musculoskeletal Disorders (WMSDs) as a structural persistence of risk [13,23,30,31]; and (iii) selected chronic conditions and the burden of benefit duration [22,25,27,33,35].

Studies on mental disorders and long-term leave show that mental illness has become a central component of the social security profile [28,34]. The association between prolonged leave and psychosocial work conditions/occupational stressors shifts the paradigm from the “sick individual” to “work as a driver of suffering and disability” [28,34]. Although not part of the reviewed corpus, international evidence from a systematic review on psychosocial occupational exposures corroborates this, demonstrating consistent associations between low social support, high psychological demands, and organizational stressors with common mental disorders, reinforcing the need for interventions focused on work organization [14,17].

The impact of the NTEP reinforces that the visibility of work-related mental disorders is an institutional phenomenon, sensitive to how occupational causality is recognized [1]. Furthermore, drawing on external literature not included in this review, recent international evidence highlights sex-specific associations between lifestyle factors, occupational exposures, and sick leave patterns [16]. For instance, while obesity may be a more prominent predictor for men, smoking behaviors often show a stronger correlation with long-term disability in women [16]. These findings suggest that prevention strategies must be tailored to these distinct profiles to effectively mitigate work disability.

The recurrence of studies on WMSDs suggests they constitute a “structural” problem within the current production model, persisting over time and across sectors [30,33]. Although not part of the reviewed corpus, international evidence on psychosocial work characteristics confirms that variables such as high psychological demands and job insecurity act as cross-cutting determinants, reinforcing the interdependence between psychosocial and biomechanical factors [12]. The complexity of these conditions is further illustrated by the fact that acute illness perceptions and previous welfare history can moderate the long-term disability trajectory, a finding from the international literature not included in this review [17], which may contribute to the structural persistence of WMSDs observed in the Brazilian productive model. Local evidence and spatiotemporal analysis reinforce that risk is not random but concentrated by productive arrangements [13,23]. This implies that generic policies often fail to address the problem where it is most intense.

The presence of studies on cardiovascular and chronic venous diseases indicates that a significant portion of temporary disability is linked to conditions with a chronic component, requiring longitudinal care and vocational rehabilitation [25,33,35]. Therefore, the impact on the system stems not only from the number of benefits but also from the duration of leave [27]. Furthermore, the complexity of the “disability event” highlights that comorbidities and underlying morbidity may not be fully captured by a single diagnostic category [22].

Based on these findings, the authors propose five priority areas for the occupational health agenda. For each area, we distinguish the evidence from the review from the authors’ recommendations:

(a) Sector-based risk management. Evidence from the review: Oliveira et al. [10] demonstrated that NTEP data can identify risk by economic activity. Authors’ recommendation: These findings support prioritizing regulatory actions based on sectoral risk profiles.

(b) Workplace mental health. Evidence from the review: The association between mental disorders and psychosocial stressors was identified in the included studies [28,34]. Authors’ recommendation: Policies should focus on work organization rather than merely individual interventions.

(c) Integrated strategy for WMSDs. Evidence from the review: The included studies confirm the structural persistence of WMSDs and their territorial concentration [13,23,30,31]. Authors’ recommendation: Prevention should combine national guidelines with regionalized implementation focused on high-incidence territories.

(d) “Sick leave trajectory” policy. Evidence from the review: The included studies demonstrate that benefit duration represents a substantial burden, with prolonged absences associated with specific conditions [22,25,27,33,35]. Authors’ recommendation: An effective agenda must balance primary prevention with case management strategies to reduce duration and recurrence, including sustainable return-to-work programs. Drawing on external literature not included in this review, integrated and early vocational rehabilitation has proven effective in improving work ability and reducing disability rates in other complex health conditions [36,37], serving as a potential benchmark for Brazilian social security reforms.

(e) Data governance and transparency. Evidence from the review: The included studies highlight the sensitivity of benefit patterns to changes in administrative procedures and assessment criteria [1]. Authors’ recommendation: Enhancing standardization and documenting operational changes increases the quality of evidence, especially during periods of disruption like the COVID-19 pandemic.

A methodological consideration regarding the scope of evidence should be noted. This review restricted search sources to indexed bibliographic databases, excluding gray literature. This decision was grounded in methodological priorities: (i) ensuring greater standardization and reproducibility of the selection criteria; (ii) facilitating the traceability of sources; and (iii) increasing comparability across studies. However, this choice entails a trade-off. Technical reports and institutional documents produced by the Federal Medical Expert Service or oversight bodies such as the Brazilian Federal Court of Accounts (Tribunal de Contas da União—TCU) may contain relevant epidemiological data and operational insights that are not captured in peer-reviewed literature. The inclusion of such documents could have enriched the analysis of institutional mechanisms, particularly regarding the operationalization of the NTEP and the impact of administrative reforms. Conversely, gray literature often lacks standardized peer review and methodological transparency, which could compromise the comparability and reproducibility that indexed databases provide. Future reviews on this topic may consider a complementary gray literature search to capture institutional perspectives, while maintaining the methodological rigor of the present approach.

### 4.1. Limitations

This scoping review has limitations. First, the restriction to quantitative observational studies excluded qualitative insights into workers’ experiences and institutional dynamics. Second, the predominance of cross-sectional studies (66.7%) restricts causal inferences and the ability to assess the long-term impact of regulatory changes. Third, the methodological heterogeneity among studies hinders direct comparability. Finally, the geographical scope restricted to Brazil limits the generalizability to other social protection systems, although international connections were established in the Discussion.

### 4.2. Contributions

The main contribution of this study is that it provides a systematic mapping of work disability in Brazil based on administrative and medical expert data. The study periodizes the institutional milestones pre-NTEP (2000–2004), post-NTEP consolidation (2005–2014), institutional reforms (2015–2019), COVID-19 pandemic (2020–2022), and post-pandemic normalization (2023–2025), offering an analytical framework for future time-series studies. These periods were defined based on key milestones in Brazilian social security policy: (a) pre-NTEP: early use of administrative records; (b) post-NTEP consolidation: stabilization of time series and expanded recognition of occupational causality; (c) institutional reforms: digitalization, benefit reviews, and regulatory changes; (d) COVID-19 pandemic: operational disruption; and (e) post-pandemic normalization: restoration of assessment flows. They are analytical categories proposed by the authors to organize the temporal discussion and correspond to the analytical framework of the broader research program of which this review is part.

The review systematizes determinants across individual, occupational, and institutional levels, providing a conceptual foundation for public policy. Finally, it translates findings into five priority areas for action, bridging the gap between scientific evidence and the concrete needs of the Brazilian social protection system.

## 5. Conclusions

This scoping review mapped and synthesized the scientific evidence on the epidemiological profile and determinants of sickness absence and work-related accidents in Brazil, with an emphasis on studies based on administrative and medical expert data from the Social Security system. The literature characterizes the profile of work disability in the country by the historical predominance of Work-Related Musculoskeletal Disorders (WMSDs), the emergence of mental disorders as an increasingly prevalent cause, and the burden of chronic non-communicable diseases associated with long-term absences. The determinants operate at multiple levels (individual, occupational, organizational, and institutional), with the Social Security Technical Epidemiological Nexus representing an important institutional mechanism that shapes the visibility and recognition of work-related illness in administrative records, although its effects cannot be fully disentangled from concurrent changes in assessment practices and administrative procedures.

Although the existing body of literature is sufficient to describe patterns and inequalities, it remains fragmented and methodologically limited by the predominance of cross-sectional studies, which restricts causal inference and the evaluation of interventions. This gap is addressed in the subsequent phase of this research program, which employs interrupted time series analysis and repeated cross-sectional designs using the full universe of Social Security medical expert records from 2020 to 2025, comparing the pandemic (2020–2022) and post-pandemic (2023–2025) periods.

Based on the review findings, the authors propose five priority areas for the occupational health agenda. We recommend (a) sector-based management of occupational risks; (b) mental health policies focused on work organization and psychosocial factors; (c) integrated territorial strategies for the prevention of WMSDs; (d) management of sickness absence trajectories (duration, rehabilitation, and return to work); and (e) data governance to ensure transparency and historical comparability.

Transcending the reactive and individualistic model depends fundamentally on strengthening the interface between evidence-based research and the formulation of integrated public policies.

## Figures and Tables

**Table 1:** Epidemiological Profile of Work Absence in Brazil.

Research Area	Key Findings	Related Studies
Work-Related Musculoskeletal Disorders	Leading cause of sick leave; high prevalence of back pain and synovitis/tenosynovitis; strong association with biomechanical overload.	Oliveira et al. [10]; Alcântara et al. [23]; Jakobi et al. [26]; Vieira et al. [30]; Andrade and Barbosa-Branco [31]
Mental and Behavioral Disorders	Third leading cause of disability benefits; post-pandemic increase; notable rise in depressive episodes and anxiety disorders.	Santos Júnior and Fischer [1]; Sá et al. [3]; Silva-Júnior and Fischer [28]; Silva-Júnior and Fischer [29]
Chronic Non-Communicable Diseases (Cardiovascular and Respiratory)	Shift from infectious causes to chronic conditions; associated with longer average sick leave durations and high costs.	Boff et al. [22]; Ildefonso et al. [25]; Morato Filho et al. [33]; Coelho et al. [35]
Institutional and External Factors	Prevalence of non-work-related benefits (B31) over work-related benefits (B91); impact of the NTEP on the characterization of the causal link.	Barbosa-Branco et al. [6]; Oliveira et al. [10] Boff et al. [22]

**Table 2:** Population Characteristics by Research Area.

Research Area	Most Affected Groups	Related Studies
Work-Related Musculoskeletal Disorders	Women; private-sector workers; ages 30 to 59; South and Southeast regions.	Alcântara et al. [23]; Jakobi et al. [26]; Souza and Santana [27]; Vieira et al. [30]; Andrade and Barbosa-Branco [31]
Mental and Behavioral Disorders	High prevalence in service industries and sectors characterized by high psychosocial stress and rigid work organization.	Santos Júnior and Fischer [1]; Sá et al. [3]; Silva-Júnior and Fischer [28]; Silva-Júnior and Fischer [29]
Chronic Non-Communicable Diseases (Cardiovascular and Respiratory)	Workers undergoing demographic transition; associated with individual and occupational risk factors.	Ildefonso et al. [25]; Morato Filho et al. [33]; Coelho et al. [35]
Institutional and External Factors	External causes result in high costs, although less frequently than WMSDs.	Oliveira et al. [10]; Boff et al. [22]

## Data Availability

The data presented in this study are available in Open Science Framework (OSF) at https://doi.org/10.17605/OSF.IO/CM95X (accessed on 18 May 2026).

## Supplementary Materials

The following supporting information can be downloaded at https://www.mdpi.com/article/10.3390/epidemiologia7040111/s1, PRISMA 2020 Checklist [38], Search Strategies by Database, Table S1. Characteristics of the 18 included studies.

## Abbreviations

The following abbreviations are used in this manuscript:

CNCDs	Chronic Non-Communicable Diseases
CNIS	National Social Security Information Registry (*Cadastro Nacional de Informações Sociais*)
DATAPREV	Social Security Information and Technology Enterprise (*Empresa de Tecnologia e Informações da Previdência*)
DeCS	Health Sciences Descriptors
JBI	Joanna Briggs Institute
MeSH	Medical Subject Headings
NTEP	Social Security Technical Epidemiological Nexus (*Nexo Técnico Epidemiológico Previdenciário*)
PCC	Population, Concept, and Context
PRISMA-ScR	Preferred Reporting Items for Systematic Reviews and Meta-Analyses extension for Scoping Reviews
SABI	Disability Benefits Administration System (*Sistema de Administração de Benefícios por Incapacidade*)
TCU	Brazilian Federal Court of Accounts (*Tribunal de Contas da União*)
WMSDs	Work-Related Musculoskeletal Disorders
