# Supplementary material for: Work-Related Illnesses and Work Disability in Brazil: A Scoping Review of the Epidemiological Profile and Determinants of Sick Leave and Occupational Accidents
Source: Epidemiologia (Basel). 2026 Aug 14;7(4):111. doi: 10.3390/epidemiologia7040111 (PMC13511866; doi:10.3390/epidemiologia7040111)
Supplement: Supplementary file 1 [file epidemiologia-07-00111-s001.zip › Search Strategies by Database.pdf]

Search Strategies by Database

Databases Searched

The following electronic databases were systematically searched:

1. PubMed/MEDLINE
2. Web of Science
3. Embase
4. Scopus
5. SciELO (Scientific Electronic Library Online)
6. LILACS (Latin American and Caribbean Health Sciences Literature)
7. CINAHL (Cumulative Index to Nursing and Allied Health Literature)
8. PsycINFO

1. PubMed/MEDLINE

(("Work Disability"[MeSH Terms] OR "Incapacidade para o Trabalho" OR "Work Ability" OR "Sickness Absence" OR "Sickness Absenteeism") AND ("Occupational Diseases"[MeSH Terms] OR "Doença Ocupacional" OR "Occupational Accidents"[MeSH Terms] OR "Acidentes de Trabalho") AND ("Social Security"[MeSH Terms] OR "Social Insurance" OR "Previdência Social") AND ("Brazil"[MeSH Terms] OR "Brasil"))

Filters: Humans; Adults (18+ years); English, Portuguese, Spanish; 2000-2025

2. Scopus

(TITLE-ABS-KEY("Work Disability" OR "Sickness Absence" OR "Sickness Absenteeism" OR "Work Ability" OR "Incapacidade para o Trabalho") AND TITLE-ABS-KEY("Occupational Diseases" OR "Occupational Accidents" OR "Doença Ocupacional" OR "Acidentes de Trabalho") AND TITLE-ABS-KEY("Social Security" OR "Social Insurance" OR "Previdência Social") AND TITLE-ABS-KEY("Brazil" OR "Brasil"))

Filters: Article; English, Portuguese, Spanish; 2000-2025

3. Web of Science

TS=("Work Disability" OR "Sickness Absence" OR "Sickness Absenteeism" OR "Work Ability" OR "Incapacidade para o Trabalho") AND TS=("Occupational Diseases" OR "Occupational Accidents" OR "Doença Ocupacional" OR "Acidentes de Trabalho") AND TS=("Social Security" OR "Social Insurance" OR "Previdência Social") AND TS=("Brazil" OR "Brasil")

Filters: Article; English, Portuguese, Spanish; 2000-2025

4. Embase

('work disability'/exp OR 'sickness absence'/exp OR 'sickness absenteeism' OR 'work ability' OR 'incapacidade para o trabalho') AND ('occupational disease'/exp OR 'occupational accident'/exp OR 'doença ocupacional' OR 'acidentes de trabalho') AND ('social security'/exp OR 'social insurance' OR 'previdência social') AND ('brazil'/exp OR 'brasil')

Filters: Article; Humans; Adults (18+ years); English, Portuguese, Spanish; 2000-2025

5. SciELO
((tw:("Work Disability" OR "Incapacidade para o Trabalho" OR "Work Ability" OR "Sickness Absence" OR "Sickness Absenteeism" OR "Capacidade para o Trabalho" OR "Absenteísmo por Doença"))) AND ((tw:("Occupational Diseases" OR "Doenças Ocupacionais" OR "Occupational Accidents" OR "Acidentes de Trabalho"))) AND ((tw:("Social Security" OR "Previdência Social" OR "Seguridade Social" OR "Social Insurance"))) AND ((tw:("Brazil" OR "Brasil")))
Filters: 2000-2025

6. LILACS
(tw:("Incapacidade para o Trabalho" OR "Absenteísmo por Doença" OR "Capacidade para o Trabalho" OR "Work Disability" OR "Sickness Absence")) AND (tw:("Doenças Ocupacionais" OR "Acidentes de Trabalho" OR "Occupational Diseases" OR "Occupational Accidents")) AND (tw:("Previdência Social" OR "Seguridade Social" OR "Social Security")) AND (tw:("Brasil" OR "Brazil"))
Filters: 2000-2025

7. CINAHL
(MH "Work Disability+" OR "Sickness Absence" OR "Sickness Absenteeism" OR "Work Ability" OR "Incapacidade para o Trabalho") AND (MH "Occupational Diseases+" OR MH "Occupational Accidents+" OR "Doença Ocupacional" OR "Acidentes de Trabalho") AND (MH "Social Security+" OR "Social Insurance" OR "Previdência Social") AND (MH "Brazil+" OR "Brasil")
Filters: Peer-reviewed; English, Portuguese, Spanish; 2000-2025

8. PsycINFO
(DE "Work Disability" OR DE "Sickness Absence" OR "Sickness Absenteeism" OR "Work Ability" OR "Incapacidade para o Trabalho") AND (DE "Occupational Diseases" OR DE "Occupational Accidents" OR "Doença Ocupacional" OR "Acidentes de Trabalho") AND (DE "Social Security" OR "Social Insurance" OR "Previdência Social") AND (DE "Brazil" OR "Brasil")
Filters: Peer-reviewed journals; English, Portuguese, Spanish; 2000-2025

Keywords Table (PCC Framework)

Population (P)	Concept (C)	Context (C)
Adult workers (≥18 years)	Epidemiological profile	Brazilian Social Security System
Formal workers (Brazilian social security system)	Determinants of sick leave	Federal Medical Expert Service
Insured population	Occupational accidents	Disability benefits (SABI/CNIS)
	Work-related illnesses	Institutional/Regulatory changes

Notes on Adaptation to Other Databases
The search strategy was adapted for each database using equivalent controlled vocabulary (Emtree for Embase, DeCS for LILACS/SciELO) and specific syntax. Boolean operators (AND, OR) were maintained across all platforms to ensure consistency in the retrieval of studies addressing the intersection of work disability, occupational health, and the social security framework.
