# Supplementary material for: Work-Related Illnesses and Work Disability in Brazil: A Scoping Review of the Epidemiological Profile and Determinants of Sick Leave and Occupational Accidents
Source: Epidemiologia (Basel). 2026 Aug 14;7(4):111. doi: 10.3390/epidemiologia7040111 (PMC13511866; doi:10.3390/epidemiologia7040111)
Supplement: Supplementary file 1 [file epidemiologia-07-00111-s001.zip › Table S1. Characteristics of the 18 included studies.pdf]

Table S1. Characteristics of the 18 included studies.

Study	Design	Population / Sector	Data Source	Period	Main Outcomes
Santos Júnior & Fischer, 2024 [1]	Ecological time series	Mental disorder benefits	DATAPREV	2003-2019	Temporal trends and NTEP impact
Sá et al., 2023 [3]	Cross-sectional	Mental disorder benefits	Social Security Yearbook	2006-2021	Temporal analysis of work incapacity due to mental disorders
Barbosa-Branco et al., 2011 [6]	Cross-sectional	Private-sector workers	DATAPREV	2008	Incidence of work and non-work disability claims
Oliveira et al., 2021 [10]	Ecological	Seven economic activities	DATAPREV	2000-2016	NTEP risk analysis
Boff et al., 2002 [22]	Cross-sectional	Beneficiaries of the General Social Security System	DATAPREV	1998	Morbidity underlying temporary disability benefits
Alcântara et al., 2011 [23]	Cross-sectional	Workers in Diamantina, Minas Gerais	National Social Security Institute	2002-2005	WMSD profile among benefit recipients
Barbosa-Branco & Ildefonso, 2012 [24]	Cross-sectional	Asthma-related benefits	DATAPREV	2008	Prevalence and duration of sickness benefits
Ildefonso et al., 2009 [25]	Cross-sectional	Respiratory disease benefits	DATAPREV	2003-2004	Prevalence of temporary disability benefits
Jakobi et al., 2013 [26]	Cross-sectional	Workers in Rondônia	National Social Security Institute	2008	Disability benefit profile
Souza & Santana, 2012 [27]	Cohort	Beneficiaries of the General Social Security System	DATAPREV	2008	Factors associated with benefit duration
Silva-Júnior & Fischer, 2014a [28]	Ecological	Mental disorder benefits	DATAPREV	2008-2011	Mental disability profile
Silva-Júnior & Fischer, 2014b [29]	Case-Control	Mental disorder benefits	National Social Security Institute	2011	Psychosocial work conditions and long-term sickness absence
Vieira et al., 2011 [30]	Cross-sectional	Private-sector workers	DATAPREV	2008	WMSD disability benefits
Andrade & Barbosa-Branco, 2015 [31]	Cross-sectional	Synovitis/tenosynovitis benefits	DATAPREV	2008	Benefit profile

Jakobi et al., 2015 [32]	Cross-sectional	Meat/fish processing workers	DATAPREV	2008	Benefit profile by economic sector
Morato Filho et al., 2018 [33]	Cross-sectional	Cardiovascular disease, Recife, Pernambuco	National Social Security Institute	2011-2015	CVD benefit profile
Silva-Júnior & Fischer, 2015 [34]	Cross-sectional	Mental disorder benefits	National Social Security Institute	2011	Psychosocial stressors and sickness absence
Coelho et al., 2020 [35]	Ecological time series	Chronic venous disease	DATAPREV	2005-2014	Temporal trends and social security burden
